# Uso de Estatinas e Hipercolesterolemia: Estão sendo Seguidas as Recomendações das Diretrizes Atuais?

**DOI:** 10.36660/abc.20210089

**Published:** 2021-04-08

**Affiliations:** 1 Departamento de Cardiologia na Santa Casa de Misericórdia de São Paulo São PauloSP Brasil Departamento de Cardiologia na Santa Casa de Misericórdia de São Paulo, São Paulo, SP - Brasil.; 2 Santa Casa de São Paulo Faculdade de Ciências Médicas São PauloSP Brasil Faculdade de Ciências Médicas, Santa Casa de São Paulo, São Paulo, SP - Brasil.

**Keywords:** Doenças Cardiovasculares, Acidente Vascular Cerebral, Infarto do Miocárdio, Mortalidade, Aterosclerose, Fatores de Risco, Inibidores de Hidroximetilglutaril-CoA Redutases, Hospitalização, Hospitais Públicos

Apesar dos avanços no tratamento das doenças cardiovasculares, o infarto agudo do miocárdio e o acidente vascular cerebral ainda são as principais causas de morte em todo o mundo.^1^

A prevenção da doença aterosclerótica coronariana (DAC), representada pelo tratamento do colesterol de lipoproteína de baixa densidade (LDL-c), é uma das principais alternativas para aumentar a sobrevida de pacientes com fatores de risco cardiovascular. Estudos de caso-controle, observacionais e genéticos confirmam a importância do nível elevado de colesterol como um dos principais fatores de risco modificáveis para doenças cardiovasculares, especialmente para DAC e acidente vascular cerebral isquêmico. A redução do LDL-c ao longo da vida tem sido associada a um menor risco de desenvolver DAC. Parece haver uma relação causal entre o LDL-c e a DAC, que é contínua e depende da magnitude da redução do LDL-c.^2^^–^^5^

Após a descoberta das estatinas em 1976 pelo bioquímico japonês Akira Endo, estudos de intervenção com essa classe de drogas mudaram a preocupação com a prevenção DAC. Atualmente, as estatinas (inibidores da 3-hidroxi-3-metil-glutaril-CoA redutase) são recomendadas por todas as diretrizes como drogas de primeira linha no tratamento farmacológico da hipercolesterolemia para prevenção primária e secundária da DAC. Esta classe de drogas atua inibindo a síntese do colesterol, aumentando assim a expressão dos receptores, resultando em maior remoção do LDL-c plasmático.^6^^–^^8^

A meta-análise mais robusta sobre estatinas avaliou dados de 170.000 pacientes em 26 estudos clínicos. Esta publicação destacou a comparação entre estatina e placebo e entre estatinas mais potentes e menos potentes. Foi observado que, com redução do LDL-c de 1 mmol/L ou 40 mg/dL, houve redução média de 22% nos desfechos cardiovasculares principais. A análise também mostrou que quanto maior a redução do LDL-c, maior o benefício obtido com o tratamento. Grandes ensaios clínicos com estatinas demonstraram que quanto maior a redução absoluta do LDL-c, maior a redução do risco relativo de eventos cardiovasculares.^5^ Até o momento, não tem sido identificado um valor de referência abaixo do qual o tratamento para redução de lipídios deixaria de promover o benefício cardiovascular; no entanto, níveis muito baixos de LDL-c foram avaliados por um curto período de tempo.^9^^–^^11^

No artigo *“Perfil de Prescrição de Estatinas e de Níveis Lipêmicos em Ambulatórios de Hospital Terciário Público”*^12^ a prescrição de estatinas foi frequente, possivelmente devido ao reconhecimento da dislipidemia como fator de risco cardiovascular relevante. No entanto, foi realizado sem uma meta específica para LDL-c, sem ajuste de dose e sem pelo menos um teste de controle anual, demonstrando que as recomendações das diretrizes não são plenamente consideradas. Além disso, mostrou que a prescrição sem avaliação do colesterol sanguíneo ocorreu predominantemente em cirurgia vascular e que a cardiologia foi a especialidade com maior número de prescrições de estatinas. Apesar disso, uma porcentagem considerável de indivíduos apresentou LDL-c acima do recomendado nas diretrizes de prevenção primária. Por outro lado, é interessante notar que, em comparação com a diretriz da AHA / ACC, a diretriz brasileira parece classificar uma proporção maior de pacientes de prevenção primária em categorias de maior risco, aumentando os critérios de elegibilidade das estatinas.^13^ Observou-se também que o uso de estatinas pelo Sistema Público de Saúde é custo-efetivo e que, entre os indivíduos tratados, 2,4% apresentavam LDL-c ≥ 190 mg/dL. Esse nível de LDL-c, superior ao registrado na população em geral, acompanhado por uma média de idade inferior à amostra total (55 ± 15 versus 63 ± 13 anos, p < 0,05), sugere a possibilidade da presença de hipercolesterolemia familiar naquele grupo. Desta maneira, seria recomendado um seguimento mais cauteloso, pois haveria maior risco cardiovascular nessa população.^14^^,^^15^

As duas estatinas usadas neste estudo, sinvastatina (78%) e atorvastatina (22%), mostraram que as concentrações de colesterol plasmático e LDL-c eram mais baixas em pacientes que recebiam prescrições da cardiologia. Portanto, seria de esperar que o cumprimento das metas preconizadas nas diretrizes, não alcançadas em grande percentual de pacientes, fosse mais alcançado por essa especialidade.

Os resultados encontrados neste estudo ilustram a necessidade não apenas de um diagnóstico laboratorial mais preciso, mas principalmente de um tratamento hipolipemiante mais eficaz. Temos dados suficientes sobre a segurança e eficácia das estatinas, inclusive para síndrome coronariana aguda.^16^

A terapia hipolipemiante mais agressiva e o diagnóstico precoce devem ser enfatizados. As estatinas continuam sendo o padrão ouro no tratamento farmacológico da hipercolesterolemia. No entanto, além de aperfeiçoar a dosagem, novos medicamentos com evidências científicas comprovadas nesse arsenal terapêutico, como a ezetimiba e os inibidores da pró-proteína convertase subtilisina/kexina tipo 9 (PCSK9), já demonstraram reduzir o risco cardiovascular com segurança.
