# Supplementary material for: Uso de Estatinas e Hipercolesterolemia: Estão sendo Seguidas as Recomendações das Diretrizes Atuais?
Source: Arq Bras Cardiol. 2021 Apr 8;116(4):742–3. [Article in Portuguese] doi: 10.36660/abc.20210089 (PMC8121391; doi:10.36660/abc.20210089)
Supplement: Translation

# Translation: Statin Use and Hypercholesterolemia: Are the Current Guidelines' Recommendations Being Followed?

**Affiliations:** 1 São PauloSP Brazil Departamento de Cardiologia na Santa Casa de Misericórdia de São Paulo, São Paulo, SP - Brazil.; 2 São PauloSP Brazil Faculdade de Ciências Médicas, Santa Casa de São Paulo, São Paulo, SP - Brazil.

**Keywords:** Cardiovascular Diseases, Stroke, Myocardial Infarction, Mortality, Atherosclerosis, Risk Factors, Hydroxymethylglutaryl –CoA Reductase Inhibitors, Hospitalization, Hospitals, Public

Despite advances in treating cardiovascular diseases, acute myocardial infarction and stroke are still the main causes of death worldwide.^1^

The prevention of coronary atherosclerotic disease (CAD), represented by the treatment of low-density lipoprotein cholesterol (LDL-c), is one of the main alternatives for increasing the survival of patients with cardiovascular risk factors. Case-control, observational, and genetic studies confirm the importance of increased cholesterol level as one of the main modifiable risk factors for cardiovascular disease, especially for CAD and ischemic stroke. The reduction in LDL-c throughout life has been associated with a lower risk of developing CAD. There seems to be a causal relationship between LDL-c and CAD, which is continuous and which depends on the magnitude of the reduction in LDL-c.^2^^–^^5^

After the Japanese biochemist Akira Endo discovered statins in 1976, intervention studies with this drug class changed the CAD prevention concern. Currently, statins (3-hydroxy-3-methyl-glutaryl-CoA reductase inhibitors) are recommended by all guidelines as first-line drugs in the pharmacological treatment of hypercholesterolemia for primary and secondary prevention of CAD. This drug class acts by inhibiting cholesterol synthesis, thus increasing expression of receptors, resulting in greater removal of plasmatic LDL.^6^^–^^8^

The most robust meta-analysis on statins evaluated data from 170,000 patients in 26 clinical studies. This publication highlighted the comparison of statins versus placebo and more versus less potent statins. It was observed that, for LDL-c reduction of 1 mmol/L or 40 mg/dL, there was an average reduction of 22% in the main cardiovascular outcomes. The analysis also showed that the greater the reduction in LDL-c, the greater the benefit achieved from the treatment. Large clinical trials with statins have demonstrated that the greater the absolute reduction in LDL-c, the greater the reduction in the relative risk of cardiovascular events.^5^ To date, no threshold has been identified below which lipid-lowering treatment would fail to promote cardiovascular benefit; however, very low LDL-c levels were evaluated for a short period of time.^9^^–^^11^

In the article “Statins Prescriptions and Lipid Levels in a Tertiary Public Hospital”^12^ the statin prescription is frequent, possibly due to the recognition of dyslipidemia as a relevant cardiovascular risk factor. However, it was performed without a specific LDL-c target, without dose adjustment, and without at least one annual control test, showing that the guidelines' recommendations are not fully considered. Moreover, it showed that the prescription without evaluation of blood cholesterol occurred predominantly in Vascular Surgery and that Cardiology was the specialty with the highest number of statin prescriptions. Despite this, a considerable percentage of individuals have LDL-c above that recommended in primary prevention guidelines. On the order hand, it is interesting to note that compared with the AHA/ACC guideline, the Brazilian guideline seems to classify a larger proportion of primary prevention patients into higher-risk categories, increasing the statin eligibility criteria.^13^ It was also noted that the use of statins by the Public Health System is cost-effective and that, among the treated individuals, 2.4% had LDL-c ≥ 190 mg/dL. This LDL-c level, higher than that registered in the general population, accompanied by a mean age lower than the total sample (55 ± 15 versus 63 ± 13 years, p < 0.05), suggests the possibility of the presence of familial hypercholesterolemia in that group. Thus, a more cautious follow-up would be recommended, as there would be a greater cardiovascular risk in this population.^14^^,^^15^

The two statins used in this survey, simvastatin (78%) and atorvastatin (22%), showed that plasma cholesterol and LDL-c concentrations were lower in patients receiving prescriptions from cardiology. Therefore, it would be expected that the achievement of goals recommended in the guidelines, not achieved in a large percentage of patients, should have been more achieved by this specialty.

The results found in this study illustrate the need not only for more accurate laboratory diagnosis, but mainly for more effective lipid-lowering treatment. We have sufficient data on the safety and efficacy of statins, including in acute coronary syndrome.^16^

More aggressive lipid-lowering therapy and early diagnosis should be emphasized. Statins continue to be the gold standard in the pharmacological treatment of hypercholesterolemia. However, in addition to enhancing the dosage, new drugs with proven scientific evidence in this therapeutic arsenal, such as ezetimibe and proprotein convertase subtilisin/kexin type 9 (PCSK9) inhibitors, have already been shown to reduce cardiovascular risk safely.
